# Structural and Electronic Properties of SnO Downscaled to Monolayer

**DOI:** 10.3390/ma15165578

**Published:** 2022-08-13

**Authors:** Adil Mubeen, Abdul Majid, Mohammad Alkhedher, ElSayed M. Tag-ElDin, Niyazi Bulut

**Affiliations:** 1 Department of Physics, University of Gujrat, Gujrat 50700, Pakistan; 2 Mechanical and Industrial Engineering Department, Abu Dhabi University, Abu Dhabi 111188, United Arab Emirates; 3 Electrical Engineering Department, Faculty of Engineering & Technology, Future University in Egypt, New Cairo 11835, Egypt; 4 Department of Physics, Faculty of Science, Firat University, Elazig 23119, Turkey

**Keywords:** SnO, layered, electronic, structural, van der waals

## Abstract

Two-dimensional (2D) SnO is a p-type semiconductor that has received research and industrial attention for device-grade applications due to its bipolar conductivity and transparent semiconductor nature. The first-principles investigations based on the generalized gradient approximation (GGA) level of theory often failed to accurately model its structure due to interlayer Van der Waals interactions. This study is carried out to calculate structural and electronic properties of bulk and layered structures of SnO using dispersion correction scheme DFT+D3 with GGA-PBE to deal with the interactions which revealed good agreement of the results with reported data. The material in three-dimensional bulk happened to be an indirect gap semiconductor with a band gap of 0.6 eV which is increased to 2.85 eV for a two-dimensional monolayer structure. The detailed analysis of the properties demonstrated that the SnO monolayer is a promising candidate for future optoelectronics and spintronics devices, especially thin film transistors.

## 1. Introduction

Two-dimensional (2D) semiconductor materials have received particular attention due to their recent applications in sensors, logic devices and, especially, in optoelectronics and spintronics [1]. Two-dimensional materials extracted from bulk solids exhibited several applications in nano-electronics devices. The worth of 2D materials having atomic thickness received serious attention after the discovery of graphene. Due to high structural and thermal stability along with exception transport properties, graphene was an initial 2D candidate for high mobility and transparent conductor devices. However, the zero band gap limited its applications in electronic and optoelectronic devices. It inspired the researchers to think about beyond-graphene 2D semiconducting materials having finite band gap for usage in photonic and spintronics devices [2]. Tin mono-oxide (SnO) having litharge (α-PbO) like structure [3] may be crystallized in the form of 2D material, which has shown device-grade potential due to its inherent p-type conduction, good stability and high carrier mobility among previously reported p-type semiconducting oxides.

Stannic oxide (SnO_2_) is an n-type semiconductor with a band gap of 3.6 eV [4], while Stannous oxide (SnO) is a famous p-type semiconductor, but can be converted from p-type to n-type when doped with Sb. It has been used as a functional material for solar cells and gas sensors, tin salts for electroplating, anode material for Lithium rechargeable batteries and especially for transparent semiconducting oxide (TSOs) [5]. Most of the TSOs present today are n-type materials, whereas p-types are just a few in numbers. Additionally, n-type TSOs present today have poor performance compared to n-type oxide semiconductors. This poor performance of p-type TSOs is mainly due to their low mobility of holes in the valence band as we compared it to the mobility of electrons in the conduction band which is relatively high. In most of the p-type TSOs, only p-orbitals of oxygen involves in the formation of VBM which limits its carrier transport [6,7,8]. However, in the case of SnO, its Sn:5s orbitals share its contribution with O:2p orbitals in the formation of VBM. This contribution of Sn:5s has enhanced significant hole mobility of SnO and can be considered a strong candidate for developing high-quality TSOs in future [9]. The SnO_2_ has already been used as saturable absorbers in ultrafast lasers due to its high electron mobility, large optical band gap (3.6 eV) and large third-order optical nonlinearities (3.8 × 10^−12^ esu) [10]. The SnO has the potential also due to high electron mobility (3.7 eV) [11] and large optical energy band gap (2.5 to 3.3 eV) [12], but the third-order optical nonlinearities have not been reported.

Results extracted from the previous theory and experiment work reveal that the origin of the p-type conductivity of SnO is the hybridization of O:2p and Sn:5s orbital in the formation of its VBM [13]. Fundamentally it is a p-type oxide semiconductor because the energy level of Sn:5s is closer to that of O:2p near the VBM and Sn:5p is near the CBM. This will cause it to generate high hole mobility. This p-type conductivity originates from the presence of Sn vacancies and oxygen interstitials [14]. Rare semiconductor oxides can develop p-n junctions and SnO is one of them. The reports based on first principles have already suggested that p-type conduction of SnO can be enhanced via structural modifications and defect engineering, including vacancies and doping [15].

The usage of SnO has an advantage over other metal oxide semiconductors due to its large electron affinity of 3.7 eV and low ionization potential of 4.4 eV. The structure of 2D SnO involves the transfer of two electrons from the p-orbital of Sn (4d^10^5s^2^5p^2^). The two electrons of the Sn:5s orbital do not participate in bonding rather they will make lone pairs that develop interlayer spacing with a Van der Waals (VdW) gap of 2.52 Å between the layers of SnO. Thus, with Sn–O–Sn sequence, it forms a layered structure with one Sn and four O atoms in pyramidal form [11]. In the formation valence band of SnO, the major contribution is from the Sn:5s and O:2p states, whereas the bottom of the conduction band is consisting of Sn:5p states [16,17,18].

Previous theoretical studies have revealed that the interaction between layers of SnO involves VdW forces which mainly control its structural and electronic properties [19]. The inter-layer lone-pair interaction plays a key role in the electronic structure of the material. In bulk SnO weak dipole-dipole VdW interlayer interaction is present in Sn–O–Sn slabs which are stacked along [001] direction. The lack of dipole–dipole lone pair interaction in its monolayer makes interesting changes in the electronic structure by increasing its band gap which will enhance its range of applications in nano-electronics devices. The modifications in an electronic structure and introduction of magnetic properties using different material engineering methods gained key research interest in designing SnO-based material having required properties [2].

While applying density functional theory (DFT) based first-principles approach, GGA exchange-correlation has been a basic choice to make accurate predictions in comparison to that of LDA and hybrid functionals (to make the calculations cost-effective). The Perdew–Burke–Ernzerhof (PBE) approach of GGA functional developed by Perdew and his coworkers is commonly used for calculating better lattice constants and surface energy especially for solids [20]. In some earlier reported predictions, GGA underestimates the energy band gap value as compared to the experimental values for the SnO. In order to realize better results of electronic properties, band gap values and Sn–Sn interactions, the DFT-D3 method is adopted along with PBE. H. Rydberg and his coworkers have suggested using the replacement of GGA-PBE functional to include VdW interactions when layered structures are simulated. This approach appeared to increase the accuracy in measurements of bond lengths, binding energies and saturation of the VdW potential at small separations of layered structures [21]. In the case of SnO monolayer, the PBE:DFT+D3 approach provided a band gap value of 2.85 eV which is closer to the experimental value of 2.7 eV in comparison to the theoretical reported value in literature with Hybrid functional (2.93 eV) [22] and calculated value (1.54 eV) with the PBE functional in this work.

This study provides an important insight into the structural and electronic properties of SnO downscaled from bulk to monolayered structure. The comparison is made between the properties of bulk and monolayer SnO materials. The mechanism is explored behind the increase in the optical band gap with the dimension changed from bulk to monolayer. The description of the band gap tuning for the material in the study may be a helpful tool to explore the structural and optical properties of other layered materials. A novel computational technique, GGA with the addition of DFT-D3, is utilized. The findings of the study are expected to provide information to utilize the material for different electronic and optoelectronic devices.

## 2. Methodology

The calculations were performed in this work to study the structural and electronic properties of the bulk and monolayer SnO. The entire calculations were DFT based and performed using the BAND module of the Amsterdam Density Functional (ADF) program [23] which was based on the scheme of a linear combination of atomic orbitals. It has always been suggested to use hybrid functional with a dispersion correction to include VdW interactions but these functionals are computationally very costly due to which alternate strategies should be explored [24]. However, to ensure cost-effectiveness, we employed GGA-PBE functional to deal with the electronic exchange-correlation interaction [25]. The GGA method has been earlier implemented to study the two-dimensional materials in DFT. In order to receive better results in terms of Sn–Sn interactions, band gap values, and electronic properties, the DFT+D3 (Grimme3) dispersion correction method was employed for the first time to study SnO. The geometry was optimized (GO) with the use of the Quasi-Newton approach and Slater Type Orbitals (STOs) were used as the basis functions. The valance configurations for the atoms Sn [Kr] 5s^2^5p^2^ and O [He] 2s^2^2p^4^ were considered, therefore, a “small” frozen core approximation was implemented. The relativistic effects “scalar” was chosen due to the Sn (with a large value of atomic number i.e., 50) was s fragment of the structure. In these calculations, we used Triple Zeta plus Polarization (TZP) basis set with a confinement radius up to 10 Bohr [26]. The lattice optimization and the positions of all atoms were relaxed until the force and total energy converged to values less than 1.0 × 10^−4^ Hartree/Å and 1.0 × 10^−5^ Hartree, respectively. The Hershfield charge analysis [27] was also implemented to investigate the transfer of charge on atoms in the structure. After a complete structure relaxation, Single Point (SP) calculations were performed to calculate the Bandstructure and Density of States (DOS).

**Figure 1:**
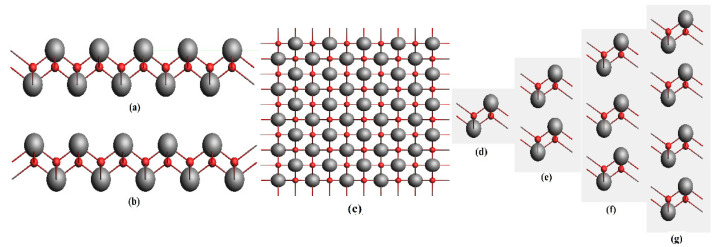
The structural view of 5 × 5 × 1 Supercell of SnO monolayer (**a**) viewed along the x-axis (**b**) viewed along the y-axis and (**c**) viewed from the top along the z-axis. The 2 × 2 unit cells of SnO in the form of a monolayer, two-layers, three layers and four layers are shown in (**d**–**g**), respectively.

SnO belonging to *P4*/*nmm* space group with tetragonal unit cells [28] forms a layered structure at normal pressures. Each Sn atom has four O neighbors which form a pyramid structure of it. Due to the presence of 5s^2^ lone pair, a weak VdW bonding also exists which makes it a layered structure [29]. The bulk and slab models of SnO were simulated in the supercell approach, thereby using the formula unit Sn_2_O_2_ with lattice constants a = 3.80 Å and c = 4.83 Å. The structure of the unit cell and 5 × 5 × 1 supercell of SnO is shown in Figure 1.

## 3. Results and Discussion

The calculations on bulk and monolayered SnO were carried out at different levels of theory and the findings are shown in Table 1.

The comparison of calculated values of formation energy found using different GGA functional suggests that PBE is suitable for SnO. The value of the band gap is also closer to that of the reported experimental value. These observations indicate the worth of PBE functional for simulating the properties of SnO, which can be further corrected by including VdW interactions for the layered material.

The calculated structural parameters and band gap along a comparison with reported values for bulk and monolayer SnO are shown in Table 2 and Table 3, respectively. GGA with PBE is adopted as XC functional to deal with the electronic exchange-correlation, whereas VdW interactions were taken into account by using Grimme’s DFT+D3 method. DFT-D3 with a suitable XC functional is most suitable to study surface adsorption, molecular crystals, solids and layered systems as well. Several improvements and perfections are observed especially in the lattice constants in solids, layer binding energy and layer separation in the layered structures system and the most prominent improvement is achieved for the dispersion-dominated systems beyond binding separation with DFT-D3 functional [30]. The calculated results are in good agreement with the reported findings obtained using PBE [5], PBE: DFT+D2 [31,32] and HSE+optB86-vdW [24].

The computed in-plane lattice parameter of monolayer SnO is 3.86 Å, Sn-O bond length is 2.26 Å, Sn-Sn length is 3.61 Å and O-O length is 2.72 Å. The findings agree with the previous DFT calculations as given in Table 2.

To investigate the structural properties of the material and understanding of the orbital contributions in the formation of its electronic structure is needed. It is observed in the previous studies that an empty state of Sn:5p was present inside the conduction band which is the indication of the Sn^2+^ oxidation state of Sn by losing two of its 5p electrons and the presence of the O-2p filled states in the locality of the valance band confirms the O^2−^ oxidation state of oxygen by gaining those two electrons. Each Sn atom by losing its two 5p electrons to the adjacent oxygen atom attained the electronic configuration Sn 5s^2^5p^0^, whereas oxygen is changed to 2s^2^2p^6^ configuration [33].

The values of charge (in units ‘e’) computed by Hirshfeld charge analysis are Sn(1) = Sn(2) = 0.35 and O(3) = O (4) = −0.35. Similarly, the charge computed by Mulliken charge analysis are Sn(1) = Sn(2) = 1.052 and O(3) = O (4) = −1.052. The overall values of Hirshfeld charge are Sn(0.7) and O(−0.7) and Mulliken Charges are Sn(2.104) and [34] O(−2.104). Sn atoms are positively charged and O atoms are negatively charged points to the charge is transferred from Sn to O atoms upon bond formation [34].

The electronegativity (E.N.) difference between comparatively electropositive Sn and electronegative O is 1.48 which predicts that the bond between SnO is polar covalent. The atomic bonding is usually examined based on ionic, covalent and van der Waals radii. The bond will be of strong covalent nature, if the sum of covalent radii is less than that of the computed bond length or if the ionic radii are greater than the computed bond length. Similarly, if the sum of covalent radii is greater than the computed Sn-O bond length between, then it is a polar covalent bond and tends to become an ionic bond. The covalent radii of Sn and O are 1.39 Å and 0.66 Å, respectively, which means that sum of covalent radii is 2.05 Å [35]. On the other hand, the ionic radii of Sn(+2) and O(−2) are 1.18 Å and 1.38 Å, respectively, which means that sum of ionic radii is 2.56 Å. The average bond lengths between Sn and O vary between 1.80 Å and 2.26 Å which pointed out that the bond Sn-O is polar covalent. The percentage ionic character was also calculated by using the Pauling formula, whose value is 43.53%. It is, therefore, concluded that the Sn-O bond in SnO is less than 50% covalent which agrees well with the Pauling formula.

**Figure 2:**
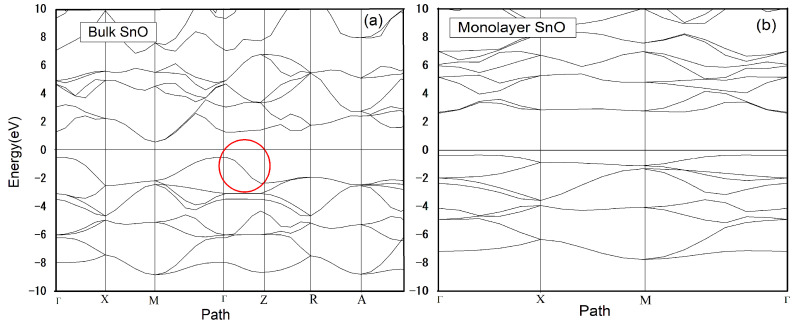
ADF calculated Band Structure calculated for (**a**) Bulk SnO and (**b**) Monolayer SnO. The Fermi level is adjusted at 0 eV.

Sn: 4d^10^ 5s^2^ 5p^2^ and O: 2s^2^ 2p^4^ are treated as valance electrons with negligible contribution of 4d^10^ and 2s^2^ orbitals in formation of SnO. O:2p orbitals provide a major contribution with a small contribution of Sn:5s orbitals in the formation of the valance band maximum (VBM) of SnO. On the other hand, Sn: 5p comprises conduction band minimum (CBM). The calculated band structure diagrams of bulk and monolayered SnO are given in Figure 2. The calculated band structure reveals that SnO bulk is a p-type semiconductor with an indirect band gap of 0.6 eV. On the other hand, a SnO monolayer is a p-type semiconductor with an indirect band gap of 2.85 eV. The VBM of bulk SnO is located at the Γ point and CBM at the M point of the Brillion zone. Whereas in the case of the monolayer, CBM is shifted towards the Γ point of the Brillion zone [2]. A small contribution of Sn:5s orbital, the major contribution of O:2p orbital in VBM and a large contribution of Sn:5p orbital in CBM. In the case of the monolayer, Sn:5s has a tiny contribution so its VBM and CBM are governed by O: p and Sn: p orbitals respectively. At the valance band, considerable band dispersion can be observed along the Γ–Z direction which is indicating a strong inter-layer interaction in bulk SnO as shown in Figure 2a.

With the electronic configuration of 4d^10^5s^2^5p^0^, the typical view of Sn can be seen after hybridization of the s and p orbitals. In this vision, Sn 5s^2^ electrons occupy an inert outer orbital that lies at one side of the Sn. As a consequence, this pair of electrons behave as a chemically inactive lone pair [36]. In the SnO interlayer, lone pair interactions are present between Sn atoms, which control its electronic structure and band gap. The interlayer Sn–Sn interaction in the bulk system is responsible for its small band gap as compared to monolayer where these Sn–Sn interactions are missing. The free surface present with lone pair states becomes the reason for band gap opening in monolayer SnO [24]. We can see a clear difference in the band gap of bulk and monolayer which is increased from 0.6 eV for the bulk to 2.85 eV for the monolayer. However, the indirect nature remains same for both.

**Figure 3:**
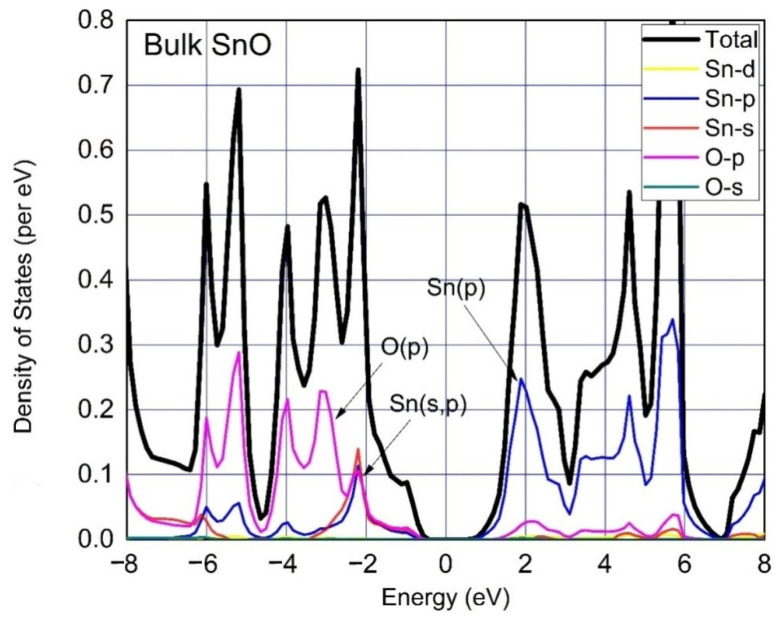
Total Density of States (TDOS) and Partial Density of States (PDOS) were calculated for Bulk SnO. The Fermi levels adjusted at 0 eV, whereas the colored lines represent states are per mentioned labels. There is a gap of 0.6 eV between CBM and VBM.

**Figure 4:**
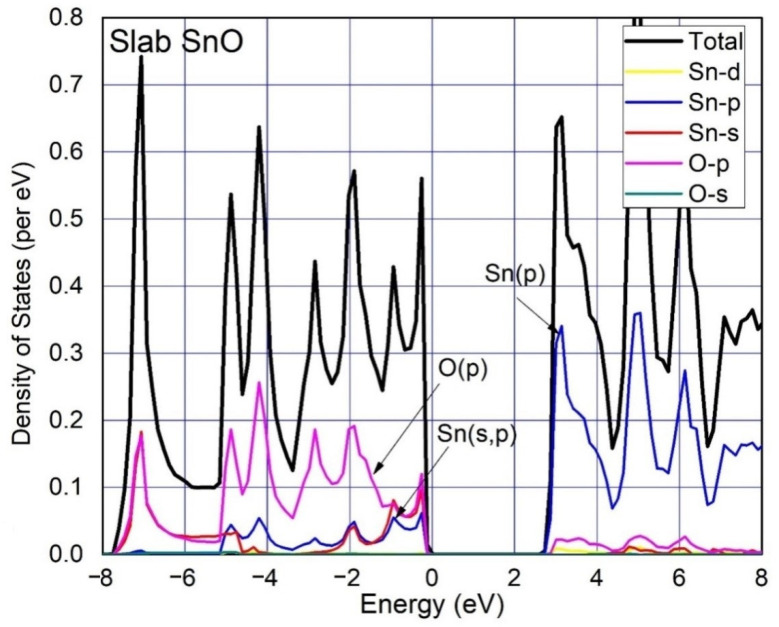
Total Density of States (TDOS) and Partial Density of States (PDOS) were calculated for monolayer SnO. The Fermi level is adjusted at 0 eV, whereas the colored lines represent states per mentioned labels. There is a gap of 2.85 eV between CBM and VBM.

The calculated total density of states (TDOS) and partial density of states (PDOS) of SnO of Bulk, monolayer, 4 layers, 3 layers and 2 layers are shown in Figure 3, Figure 4 and Figure 6, respectively. The spin polarization is completely absent, and the magnetic moment is zero, which shows that pure SnO is a non-magnetic semiconductor. The observations point to the widening of the band gap of SnO from bulk to monolayer. PDOS results reveal orbital contributions in the formation of the electronic structure of SnO. A large contribution of O:2p orbital in VBM and Sn:5p orbital in CBM for both bulk and monolayer are obvious from the DOS. VBM of SnO is governed by O: p orbitals, whereas its CBM is governed by Sn:p orbitals. The O:2s states are completely filled and do not contribute to the formation of VBM as well as CBM [37]. At the VBM a large density of Sn:5s and O:2p states is considered to be the origin of high hole mobility in bulk as well as layered SnO [13,24]. An insignificant portion of the Sn: d orbital can be seen which clarifies its negligible contribution to the formation of VBM and CBM. We can also see the contribution of Sn:5s orbital in the formation of VBM of the bulk material in Figure 3, but this contribution is relatively small compared to the O:2p as shown in Figure 4 due to the minor Sn–Sn interlayer interactions in the monolayer.

A chemically inactive lone pair made by Sn:5s electrons away from the Sn–O–Sn layer has the main role in the structural stability of SnO [38]. The lone pair states present on the surface are the reason behind the band edges of the monolayer. These lone pairs are more stable and almost isolated in the case of monolayers, whereas their isolation and stability decrease with an increase in the number of layers and end in the bulk SnO. In bulk SnO, this lone pair breaks out and gives a small band gap compared with the monolayer SnO [24].

**Figure 5:**
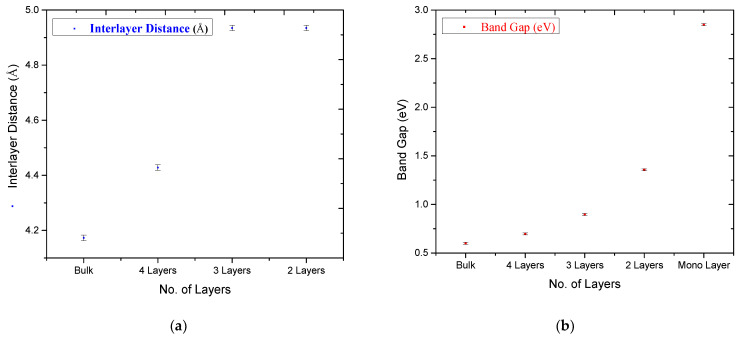
(**a**) Inter layer distance (**b**) Band gap, as a function of the number of layers of SnO.

A decrease in Sn–Sn distance causes to decrease in the band gap. This variation in band gap for several layers can be viewed from the data given in Table 3 and also in Figure 5. An increase in the band gap with a dimensional reduction from bulk to the surface has been reported in earlier experimental and theoretical studies. The reduction in band gap values for bulk to single layer structural modification is observed in experimental studies of band gap engineering in ZnO thin film [39] and MoS_2_ optical investigation [40], whereas it has been also predicted in the theoretical studies of WS_2_ [41] and hexagonal 2D semiconductors [42]. The experimental study on SnO downscaled to monolayer has not been reported yet in literature. Our theoretical results are setting a simple and straight strategy to control the band gap of SnO. In Bulk structure, every layer of Sn experiences forces from its upper side as well as from the lower side. These forces are the reason to shrink the band edges as well as the band gap up to 0.6 eV of bulk SnO.

**Figure 6:**
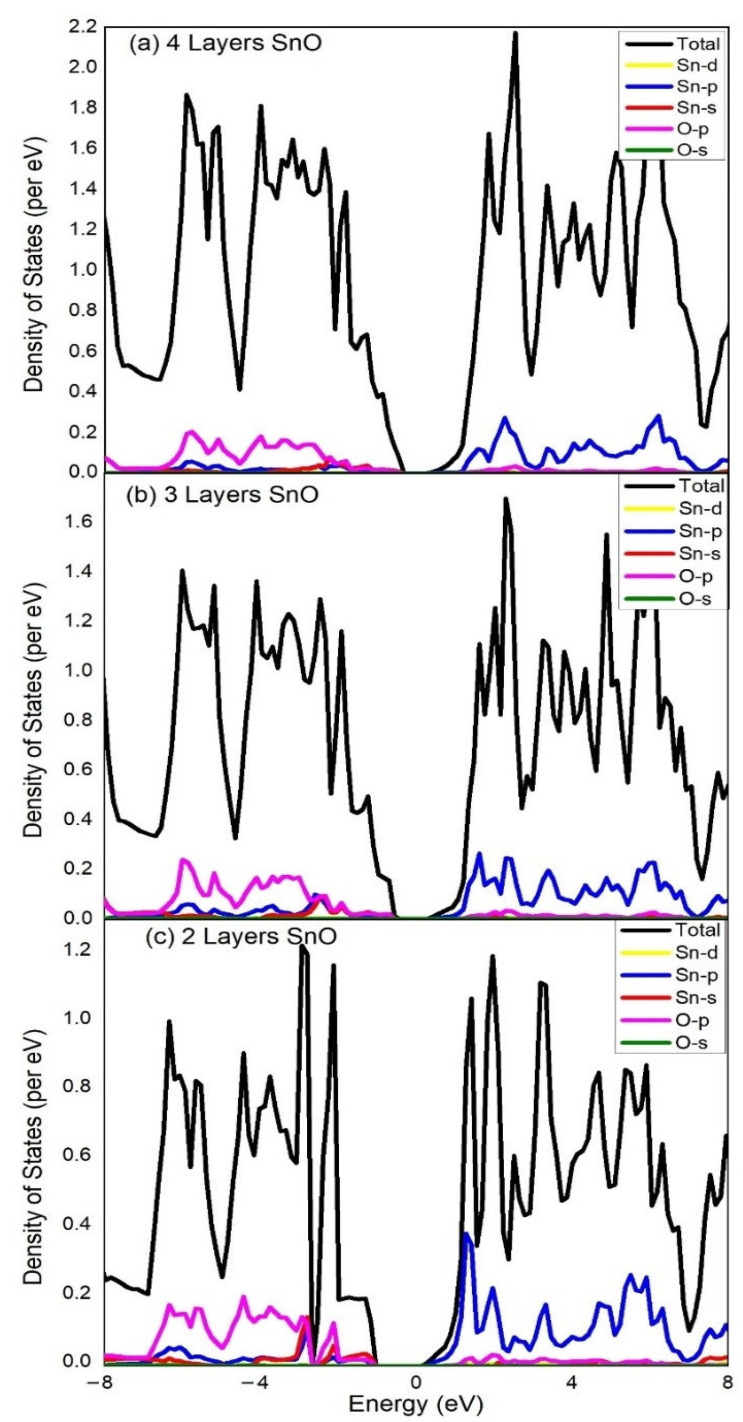
Total Density of States (TDOS) and Partial Density of States (PDOS) were calculated for (**a**) 4 layers, (**b**) 3 layers and (**c**) 2 layers of SnO. The Fermi level is adjusted at 0 eV, whereas the colored lines represent states per mentioned labels.

A decrease in the number of layers will reduce these forces and, eventually, an increase in the band gap can be observed clearly in Figure 5. The comparison of calculated TDOS and PDOS calculated for 4 layers, 3 layers and 2 layers of SnO is given in Figure 6. It shows the gradually decreasing orbital contribution of Sn:5s from 4 layers to 2 layers of SnO in the formation of VBM. It happened due to the drop in Sn–Sn interlayer interactions which gradually decrease from bulk to monolayer SnO. There is a gap of 0.697 eV, 0.89 eV and 1.35 eV between CBM and VBM for 4 layers, 3 layers and 2 layers, respectively. As the number of layers is diminishing, the band gap starts increasing in the same way and gets its maximum value for the monolayer where we have just one layer of SnO. This band gap engineering tool is incredibly effective to control the structural, electronic and optical properties of future smart materials. The free surface present with lone pair states becomes the reason for band gap opening in monolayer SnO. Due to its optical transparency, 2D SnO is favorable for future optoelectronics devices. Being one of the semiconductor oxides which have optical transparency, high carrier mobility and ambipolar conductivity, 2D SnO is considered the most suitable material for future thin film transistors.

**Figure 7:**
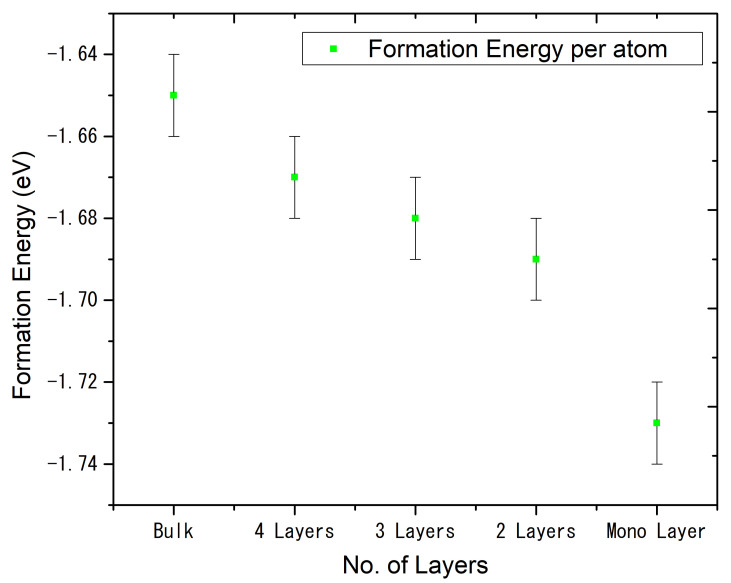
Formation Energy per atom as a function of several layers of SnO.

The SnO stability with different layer sizes is also studied in this work. The calculated values of formation energies of the monolayer, 2 Layers, 3 Layers, 4 Layers and Bulk SnO are −1.73, −1.69, −1.68, −1.67 and −1.65 eV per atom, respectively, which are plotted in Figure 7. It can be seen that the formation energy hence the stability of layered structures is higher for the layers than the 3D bulk which points to preferential growth of layeredness SnO. The results are showing that when we move from bulk to the monolayer SnO, the formation energy decrease, whereas the band gap energy value increases. With the dimensional reduction from bulk to monolayer, the Van der Waals interlayer interaction forces in the monolayer will disappear [43]. It points to more structural relaxation and a slightly larger lattice parameter of the monolayer as compared to that of the bulk as shown in Table 2, also less value of formation energy for the monolayer as shown in Figure 7. The rise in band gap with a decrease in the number of layers points out the quantum confinement effect [44]. In the case of a monolayer, the particle size is too small to be comparable to the wavelength of the electron and at the nanoscale, the decrease in confining dimension makes the energy levels to discrete and widens the band gap; hence, increases in the band gap energy. The findings predict a high likelihood of growth of SnO in the form of monolayers which indicates the availability of the material for several relevant device-grade applications.

## 4. Conclusions

PBE functional including DFT+D3 is suitable to simulate 2D SnO which is a layered material governed by Van der Waals interactions. A comparative study from bulk to monolayered SnO was made to investigate the structural and electronic properties of the materials. An increase in band gap is observed when we move from bulk to monolayer through different layer sizes of SnO. The interlayer lone pair interactions are present between Sn atoms of SnO which control its electronic structure. The interlayer Sn–Sn interaction in a bulk system is responsible for its small band gap (0.6 eV) as compared with the monolayer band gap (2.85 eV) where these Sn–Sn interactions are missing. The growth of SnO in the form of monolayers is energetically favorable when compared with that of the bulk. Hence, the 2D SnO is considered favorable for future optoelectronics and thin film transistors devices due to its optical transparency and ambipolar conductivity.

## Figures and Tables

**Table 1:** A comparison of formation energy and band gap of monolayer SnO was calculated using different GGA functional.

Functional	Formation Energy (eV)	Band Gap (eV)
Experimental	-	2.5 to 3.3 [12]
PBE	−19.87	1.544
BP86	−17.55	0.319
BLYP	−15.90	0.103
mPBE	−17.54	0.185
mPW	−17.52	0.24
OLYP	−15.99	0.22
OPBE	−17.62	0.358
PW91	−18.99	0.274
revPBE	−16.71	0.104
RPBE	−16.51	0.058

**Table 2:** A comparison of the calculated values with the literature values for (a): bulk SnO (b): SnO monolayer.

**(a)**					
**Parameters**	**PBE: DFT+D3**	**PBE** [12]	**HSE** [12]	**HSE+optB86-vdW** [24]	**Exp.** [12]
a (Å)	3.859	3.860	3.794	3.847	3.801
b (Å)	3.859	3.860	3.794	3.847	3.801
c (Å)	5.533	5.042	4.912	4.823	4.835
Volume (Å)^3^	82.4	75.14	-	-	69.85
Sn-O (Å)	2.266	2.252	-	-	2.222
Sn-Sn (Å)	3.619	3.582	-	-	3.539
O-O (Å)	2.729	2.730	-	-	2.688
Band Gap (eV)	0.6	0.4 [24]	0.8 [24]	1.1 [24]	0.7 [24]
**(b)**					
**Parameter**	**PBE: DFT+D3**	**PBE** [5]	**PBE:DFT+D2** [31]	**PBE: DFT+D2** [32]	**Exp.** [12]
a (Å)	3.861	3.866	3.83	3.86	3.801
b (Å)	3.861	3.866	3.83	3.86	3.801
Volume (Å)^3^	14.9	-	-	-	-
Sn-O (Å)	2.266	-	1.805	2.25	-
Band Gap (eV)	2.85	-	3.281	2.7	2.5 to 3.3

**Table 3:** Structural and electronic properties were calculated for bulk and layered SnO up to 4 layers. The inter-layer and intra-layer bonding parameters are given.

Parameter	Monolayer	2 Layers	3 Layers	4 Layers	Bulk	
a (Å)	3.86	3.88	3.87	3.87	3.86	
b (Å)	3.86	3.86	3.87	3.87	3.86	
Volume (Å)^3^	14.9	15	15	15	82.4	
**Intra-layer**						
Sn-O (Å)	2.267	2.266	2.266	2.265	2.266	
Sn-Sn (Å)	3.619	3.612	3.612	3.612	3.616	
O-O (Å)	2.731	2.74	2.74	2.74	2.73	
**Inter-layer**						
O-O (Å)	-	4.945	4.945	4.944	4.944	
Sn-Sn (Å)	-	4.935	4.935	4.428	4.173	
Band Gap (eV)	2.85	1.356	0.896	0.697	0.6	

## Data Availability

Data will be made available on request.
